# The Hospital Treatment of Infectious Cases

**Published:** 1909-06-26

**Authors:** A. Knyvett Gordon

**Affiliations:** Medical Superintendent of Monsall Hospital, Lecturer on Infectious Diseases in the University of Manchester


### SPECIAL ARTICLES.

### THE HOSPITAL TREATMENT OF INFECTIOUS CASES.

By A. KNYVETT GORDON, M.B.Cantab.; Medical Superintendent of Monsall Hospital, Lecturer on Infectious Diseases in the University of Manchester.

In any large general or special hospital problems not infrequently arise in the treatment of patients who, for one reason or another, are, or may become, a source of infection to others, and whom it is not always possible or desirable to transfer to another institution. In this paper I purpose dealing with the methods which have been adopted from time to time to meet these difficulties.

A patient may require to be "isolated" either because he may be infectious to others in the same institution, or because he may himself contract infection from them. It is only in fever hospitals, however, that difficulties occur with regard to the latter, and I need not consider these now. The precautions that may be taken for segregation of patients who are themselves infectious are of two kinds, structural and personal; and though these may be, and often are, combined in any given instance, yet they depend for their efficacy on such different conceptions of the nature of infection that it will be well to consider them separately. Often, though quite erroneously, they are regarded as being mutually interchangeable.

The separation of patients by structural methods is the oldest plan, and was for many years the only one employed. It was then thought that infection is disseminated mainly, if not entirely, by aerial convection, that it suffices, therefore, to place the patient in an isolation ward, and that it is then unnecessary to adopt any special precautions in nursing him, or in dealing with the utensils employed for his treatment. It was not uncommon, therefore, to find the isolation block of a general hospital consisting of a series of small rooms all served by the same staff, with one room or kitchen attached where the appliances for all the patients were "washed up" together. A modern development of this is the cubicle system whereby one large ward with glass partitions between the beds is substituted for the several small wards. Architecturally, these cubicles have some advantages over the small wards, and also some rather glaring disadvantages. I do not purpose discussing these here, but it is necessary to point out that the cubicle system in itself affords structural separation only, which is in this respect neither more nor less effective than its predecessors, though it may have some advantages in point of convenience.

Provided that there is no overcrowding of the patients in the ward—and for this purpose we may take 2,000 cubic feet of space for each patient as the irreducible minimum—it is not nowadays believed that infection does spread by aerial convection, except in the case of small-pox, chicken-pox, and, perhaps, measles; and even in these we do not consider this to be the main factor, which is almost invariably to be found in the hands, clothing, and instruments of the attendants and in the clothing (including bed-linen) and utensils which have been in contact with the patient. Consequently, when an isolation ward or cubicle is available the patient can well be placed therein, but the personal precautions must be adopted as well, with just the same strictness as in the general ward. In the vast majority of diseases other than those just named, structural separation is quite unnecessary, and its employment tends to neglect of the essential attention to details in the nursing. It is a physical impossibility for any one nurse to supervise others under her, in a group of small wards.

It will be well, then, to consider what precautions should be adopted in the case of a patient admitted to a general ward, in order to prevent the dissemination of infection from him. Given the due observance of these, it is safe, in my opinion, to admit to the general medical or surgical wards such diseases as diphtheria, typhoid fever, erysipelas and its allies, and even scarlet fever and whooping-cough, though it would be well to exclude variola, varicella and measles. There is, however, one structural essential which has been mentioned, namely, that the cubic space for each patient shall be ample and the ventilation free. The reason why such diseases as scarlet fever and whooping-cough are apt to spread in a general hospital is not only because the personal precautions which I shall describe are not adopted, but because so often the pressure on accommodation leads to diminution of the cubic space below the requisite minimum of 2,000 feet per patient.

Coming now to the details of the personal precautions: the first essential is the erection round the patient's bed of a "barrier," the main purpose of which is to act as a label and thus show that a certain ritual must be adopted in the nursing of the patient inside it. It should, however, be an efficient label, and consequently I do not consider that the mere placing of a coloured cord round the bed is sufficient. This may be enough for the physician, but it presupposes the same care and knowledge on the part of a newly joined probationer or a junior nurse, who may be just as potent a factor in spreading infection as the medical man.

After trial of many devices, I have for the last three years adopted a circle of screens, about 3 ft. high, covered with sheets wetted with a weak disinfectant solution. It is not suggested that the solution kills the infecting microbes, but a wet sheet probably arrests a certain amount of dust which would otherwise disseminate infection amongst the other patients in the ward. Moreover the labour of keeping the sheets wet may afford a useful lesson in hygiene for the junior nurses.

Beside the patient's bed, inside the barrier, is a locker which is preferably made of metal covered with the same kind of enamel as is used for the familiar cups and plates; this can be easily kept clean, and its surface is far superior to that afforded by any kind of paint. It is also a convenience, especially in surgical cases, to have a couple of glass shelves fixed on the wall over the bed. In the locker or on the shelves the utensils for the exclusive use of the patient are kept.

On the screen a washable gown for the use of the nurse is hung, and also a card on which her instructions are clearly set out. These will naturally vary somewhat in different institutions, but those in use at Monsall Hospital may perhaps be given as a type. They are as follows :

(1) Rubber gloves are to be worn by the nurse for all manipulations connected with the case, including the handling of clothes. The gloves are to stand in a bowl of 1 in 400 izal solution.

(2) The following utensils are to be marked and kept on the glass shelves or in the locker: Spatula, nozzles, clinical thermometer—all to be kept immersed in izal solution. At least two bowls. All feeding utensils.

(3) A plentiful supply of wet swabs is to be kept on the locker, together with a bowl containing izal solution to receive these when used. Handkerchiefs are not to be employed.

(4) No toys or books that have once been used inside the barrier are to be taken outside it except to be destroyed.

(5) In every case a piece of jaconet is to be placed on the pillow-slip, and over this a piece of muslin; the latter is to be burnt whenever it becomes soiled.

(6) An overall is to be worn by the nurse whenever either the patient or his clothes are handled. This is to be kept inside the barrier.

It will be seen that these rules aim at safeguarding the hands and clothing of the nurse and at distinguishing the utensils and appliances for the treatment of the particular patient. The next essential is that means shall be taken to prevent these latter coming in contact with, and thus infecting, those employed for other patients. To this end it is not sufficient that they should only be labelled, but they should be treated in a different manner, and, where the structural arrangements of the ward permit, it is best that every appliance used for a "barriered" case should be sterilised by boiling in a large vessel, and not merely be "washed up" in the ward kitchen or lavatory. Where this is not possible—and for some unexplained reason it is not always considered necessary that a steriliser should be provided for a "medical" ward—it will be necessary for the responsible nurse to see that all appliances that have been used for the other cases are removed from the duty room before those from the barriered case are brought there, and that they are cleansed by the nurse and not by the wardmaid. The most important utensils to be considered are cups, feeders, and spoons; and the practical point is really to treat these as surgical instruments.

There should not be any difficulty in the use of the rubber gloves by the nurses, or in the preservation of the gloves themselves. At Monsall they have been in use by the nurses for seven years, and the system has worked without a hitch. The gloves should be of the quality styled "extra thick," and hitherto those made in America have worn the best. They are a little more costly than the English ones, but they stand repeated boiling very much better, and do not become brittle in use.

It is essential that the nurses should understand that the bare hands cannot be made sterile, or in any way "safe," by immersing them in a disinfectant solution, or by holding them under a spray or tap. Most nurses in the average ward have to "disinfect" their hands so frequently that if gloves are not employed the skin either becomes chapped or the process is shirked. When gloves are used, however, the texture of the skin is improved, while the gloved hands can be rendered sterile, as I have shown by experiment, by holding them under a spray or running water from a tap for one minute.

It may be well to state briefly the results of this system of isolation. At Monsall Hospital the greater part of the patients are children of the age at which the susceptibility to infection is at its highest; moreover, the fact that they are already suffering from one infectious disease considerably increases that susceptibility. While the system to which I have referred has been of gradual growth, in so far as the details are concerned, the main features—namely, the barrier itself and the use of rubber gloves by the nurses—have been in existence for six years. The other details have remained unaltered for the last year.

On only one occasion has infection been carried from a "barriered" case to others, and then, owing to a misunderstanding, there was a delay in the isolation of the patient, who, incidentally, was suffering from measles. In every other case, either the patient himself or the others in the ward have been effectively protected from infection. My experience does not enable me to speak as favourably about those treated in small "isolation" wards. In fact, the barrier was introduced because structural separation so often failed. Consequently I feel justified in recommending the adoption of the "barrier" system in other hospitals whose circumstances render it impossible or inadvisable to transfer the patient to the isolation hospital of the neighbourhood.

